# Good response to rectal diazepam in refractory cases of cyclic vomiting: A case‐series and review of the literature

**DOI:** 10.1002/ccr3.8109

**Published:** 2023-11-20

**Authors:** Mansoureh Togha, Mahsa Babaei, Melika Jameie

**Affiliations:** ^1^ Neurology department, Sina Hospital, School of Medicine Tehran University of Medical Sciences Tehran Iran; ^2^ Headache department, Iranian Center of Neurological Research, Neuroscience Institute Tehran University of Medical Sciences Tehran Iran; ^3^ Iranian Center of Neurological Research, Neuroscience Institute Tehran University of Medical Sciences Tehran Iran

**Keywords:** cyclic syndrome, diazepam, migraine, nausea, vomiting, VomitingCinnarizine

## Abstract

**Key Clinical Message:**

Although increasing in number, cases of CVS are being frequently misdiagnosed and many are refractory to the available treatments. This paper draws attention to a timely consideration of this disorder upon suspicion and proposes rectal diazepam and cinnarizine as highly effective treatments in refractory cases of CVS.

**Abstract:**

Cyclic vomiting syndrome (CVS) is a set of recurrent episodic attacks of nausea and vomiting. This is a migraine‐related disorder that mostly affects children. Several medications have been recommended for abortive and prophylactic treatment. Unfortunately, in some cases, the treatment is not completely effective and affects the quality of life of the sufferer. In this paper, we report on two cases of children experiencing refractory CVS attacks who were not responsive to recommended medications for acute phase and prophylaxis. This account highlights the efficacy of rectal diazepam for the acute phase of CVS and cinnarizine, an anti‐migraine and anti‐histamine agent, for prophylaxis of further attacks.

## 1 INTRODUCTION

Cyclic vomiting syndrome (CVS) is a set of recurrent episodic attacks of nausea and vomiting.^1^ The diagnostic criteria includes: (a) at least five attacks of intense nausea and vomiting in a predictable and stereotypic pattern that occur at least four times per hour; (b) the episodes last for more than 1 h up to 1 week; (c) attacks occur one or more weeks apart; (d) there is complete symptom resolution between attacks; (e) the condition cannot be not explained by another disorder.^1^ This disorder usually proceeds in the following phases: prodromal, vomiting, recovery, and symptom‐free.^2^

CVS was first described by Heberden in 1806^3^ and currently has an estimated prevalence of 1.9% and 2.6%.^4^, ^5^, ^6^ This disorder was previously considered to only affect children and later was determined to also affect adults.^1^ Although the precise etiology of CVS is unknown, studies have suggested mitochondrial disturbances and autonomic dysfunction as the underlying pathophysiology of the disorder.^7^ According to the literature, factors such as stress, excitement, infection, and menstrual periods can trigger CVS onset.^8^ Moreover, a panic attack,^8^ anxiety,^8^ marijuana use,^9^ diabetes mellitus,^9^ and irritable bowel syndrome are common among CVS patients.^9^

CVS is typically considered as a migraine‐related disorder.^1^, ^4^ A high proportion of CVS patients also have migraine headaches^10^, ^11^ and children with CVS are highly likely to develop migraine headaches in adulthood.^4^, ^12^ A family history of migraine headache is highly prevalent among CVS patients, which suggests their similar pathophysiology.^13^

A diagnosis of CVS is made upon careful consideration of its alarming symptoms by taking a comprehensive patient history, physical examination, lab tests, and imaging for exclusion of other disorders, followed by fulfillment of the CVS diagnostic criteria as mentioned above.^1^, ^14^ The diagnosis is often delayed because the findings are usually non‐specific and patients may undergo unnecessary diagnostic tests and interventions prior to a final diagnosis of CVS.^7^ Moreover, there is a high chance of misdiagnosis in CVS cases; therefore, knowledge should increase in this area.^10^

Management of CVS includes identification and avoidance of its triggers, treatment with prophylactic agents to prevent attacks, improvement of the acute phase with abortive medication, and continued medical and psychological support care to the patient and the family.^7^, ^14^ The prognosis of CVS is varies greatly among individuals.^15^ Generally, in 60% of patients, there is a complete symptom resolution within 1 year of the onset of treatment.^16^

## 2 CASE PRESENTATION

### 2.1 Case 1

A 12‐year‐old female presented to the emergency department with an episode of disabling nausea and vomiting, which was followed by abdominal pain. The duration of each episode was 2–3 days and the frequency of attacks in each episode was three to four times per hour in the initial hours and then every 1–2 h. The vomiting was non‐projectile, non‐bilious, and included ingested food. The patient also reported concomitant stomachache associated with each episode. The patient had reported experiencing similar episodes since the age of 4 years. The episodes had been occurring every 5 or 6 months, but had increased within the last 7 months to two to three times per month.

Each attack was followed by intense abdominal pain and the episode lasted for 3 days. There was no preceding stressor or triggering factor recognized for the attacks and there were no prodromal symptoms associated with the episodes. The emetic phase was generally similar to the latter episode described above. Insomnia was usually associated with the episodes and, in the most recent 7 months, dizziness was also associated with the episodes. The patient did well between episodes and had no history of medical disorder; however, there was a history of migraine headaches on the mother's side of the family. The patient otherwise showed normal developmental status.

The patient had presented and been admitted to the emergency ward in the past and had visited in gastrointestinal and neurological clinics multiple times. She was investigated thoroughly for a possible gastrointestinal or neurological disorder and underwent routine tests for celiac disease with negative results. In the past, preventive medication with nortriptyline and propranolol had been prescribed, but they had not been effective in preventing further attacks. During past attacks, the patient also had received intravenous acetaminophen and anti‐emetic agents, which only slightly improved the symptoms.

Upon referral to our headache clinic, the patient's physical examination and neurological evaluations were normal as well as her mental status. Brain MRI and EEG revealed normal findings. Meticulous investigations were performed for assessment of all possible gastrointestinal, neurological, metabolic, immunological, and psychiatric disorders. The examinations and test results were reported to be normal. After exclusion of all these possibilities, we considered CVS because her presentations fulfilled the diagnostic criteria.^1^

This is noteworthy that abortive treatment using different oral and parenteral NSAIDS, acetaminophen, and antiemetics had been administered previously, but none were able to effectively abort the symptoms. Prophylactic drugs such as sodium valproate, topiramate, propranolol, amitriptyline, flunarizine, and pizotifen had not been helpful in decreasing the attack frequency, duration, or severity in the year prior to our assessment.

The abortive therapy prescribed was 10 mg of rectal diazepam at the onset of the attack, which decreased the duration and severity of the attack up to 50%. The prophylactic treatment prescribed was 75 mg of cinnarizine every night. This alone was able to decrease the frequency of attacks by 50% after about 1 month of usage, but no change in the severity or duration of the attacks occurred. In addition, 300 mg of riboflavin and 300 mg of magnesium per day were initiated 3 months after the onset of treatment. The patient reported no attacks for 4 months after beginning these medications and the duration and severity of subsequent attacks decreased up to 30%.

### 2.2 Case 2

A 14‐year‐old male presented to our tertiary clinic with recurrent episodes of severe non‐bilious vomiting that had begun at the age of five and recurred every 2–3 months with each episode lasting for 1–2 days. During each episode, the attacks of nausea and vomiting occurred every 20 minutes and were associated with loss of appetite. The patient could only drink water. The attacks were accompanied by severe stomachache.

The patient reported that the main triggers of the episodes were usually stress and sleep deprivation and the episodes usually occurred during or after travel or a change in life habits, including diet and sleep routines. Between episodes, he reported being symptom free and without any gastrointestinal issues. There was a positive history of migraine headaches in the family of the patients' mother, but the patient had no past medical conditions other than the episodes described. He had previously been thoroughly assessed by physical examination and investigations that included gastrointestinal evaluations, EEG, MRI, lab assessments of general conditions, such as renal and hepatic tests, and metabolic studies, all of which resulted in normal findings. The only finding was sporadic non‐specific sharp waves on the EEG. An initial diagnosis of abdominal epilepsy had been made and medication with antiepileptic agents were initiated, but none were able to improve the symptoms except for sodium valproate, which was initiated at the age of eight and decreased the frequency of the episodes. A summary of the clinical findings and evaluation of the cases is presented in Table 1.

**TABLE 1:** Summary of the clinical examination, lab test and imaging findings of the patients.

Summary table	Chief complaint	Physical and neurological examination	Lab tests	Brain MRI	EEG
Case 1	Frequent episodes of nausea and vomiting	Normal	Normal	Normal	Normal
Case 2	Frequent episodes of nausea and vomiting	Normal	Normal	Normal	Sporadic non‐specific sharp waves

At the age of 10 a diagnosis of CVS was made and he was prescribed migraine‐prevention medications, including propranolol and amitriptyline, which were unable to improve the symptoms. Moreover, injection of acetaminophen and NSAIDs during the attacks were ineffective in aborting the symptoms. Anti‐emetic agents, including ondansetron, brought little relief from the symptoms.

Interestingly, medication with 75 mg of cinnarizine every night for 2 years dramatically decreased the severity of the attacks as well as the frequency of the episodes by 70%. The IV injection of 10 mg of diazepam considerably improved the nausea, vomiting, and stomachache attacks. Rectal diazepam at the same dosage also alleviated the symptoms. At the age of 13, the patient experienced the onset of typical migraine headaches with a frequency of once or twice a month.

A summary of the clinical, laboratory, and imaging findings of the two cases have been reported in Table 1.

## 3 DISCUSSION

Considering the prevalence of CVS in the population and the large number of missed diagnoses of CVS, further investigation is required to determine more about the nature and pathophysiology of this disorder and develop better diagnostic and therapeutic approaches for both pediatric and adult populations.^8^ Because adults with CVS develop more challenging presentations and require different therapeutic approaches, more detailed recognition and comparison of the different features of this disorder among pediatric and adult patients can aid in the development more targeted and more effective diagnostic and therapeutic guidelines.^8^

According to the literature, 43%–93% of CVS patients have recognizable prodromal symptoms; however, our cases reported no prodromal symptoms associated with their CVS episodes.^8^, ^16^ Consistent with our cases, the emetic phase is usually characterized by persistent nausea and vomiting with a frequency of every 2 h to 20 times per hour that are associated with the other symptoms.^8^ Studies have shown that, at the time of diagnosis, the frequency of the vomiting episodes was at least once a month in 41% of patients, one every 3 months in 37%, and at intervals of greater than 3 months in 22%.^16^ The intervals between the episodes in our cases were every 1–3 months and the length between episodes increased significantly over time after administration of appropriate medications.

Similar to our cases, other studies have reported that 58% of CVS cases experienced abdominal pain during the emetic phase.^8^ They have also reported low‐grade fever, neutrophilia, tachycardia, and hypertension as being associated with the emetic‐phase symptoms.^8^ Intense distress and intense thirst during the emetic phase can cause mentally normal patients to behave abnormally and demandingly, for example, drinking large amounts of water to ease the attacks, which sometimes has been mistaken as being psychotic or bulimic.^8^ Our cases reported dizziness and intense thirst during the emetic and recovery phase. Studies have found that the vomit usually contains food material, bile, and blood.^8^ Our cases reported vomit that was non‐bilious, non‐bloody, and contained food material.

Our patients reported attacks of uniform length, which is consistent with the findings of Fleisher et al.^8^ who reported that 85% of their CVS patients reported attacks of uniform length and 15% had attacks of varying length. Studies have suggested that each episode usually lasts 2 days for youths and 3.8 days for adults with CVS.^10^

Interestingly, most CVS patients (82%) in one large study reported that the episodes began between midnight and noon, 11% reported experiencing the onsets in the afternoon or evening, and 5% reported onsets at any time of the day.^8^ Our cases were similar to the third group, with the onset of attacks onset reported to be at any time of day. This could be due to an increase in the strength of the trigger factors, including stress during the day and a lack of sleep at night, both of which are known triggers.

Studies have reported that the age at onset of CVS ranged from 2 to 49 years.^8^, ^16^ As in our cases, it generally required 2.6 and 7.9 years from the onset of episodes to the diagnosis of CVS.^10^ This caused years of unnecessary suffering from untreated attacks as well as repeated and inconclusive invasive diagnostic interventions^16^ and was consistent with our cases.

The duration of CVS disorder has been reported to be less than 1 year to over 43 years in previous studies.^8^ Our cases reported decreases in the frequency of the episodes, but not complete resolution of the symptoms.

As stated, several comorbidities have been recognized for CVS^8^, ^9^, ^10^; however, our Case 1 reported no comorbidities and Case 2 reported late‐onset migraine headache. Interestingly, the presence of migraine headache in the family of the mothers of both cases was in line with findings from previous studies and supports the notion that that CVS shares a pathophysiology that is similar to migraine headaches.^13^ Our findings were consistent with those of previous studies which found that 57%–66% of CVS cases had first‐degree and/or second‐degree relatives with migraine headaches or its variants.^8^ Of these, 83% of the family history was matrilineal, 13% patrilineal, and 4% undetermined.^8^ A positive family history of migraine was more often observed among pediatric cases of CVS.^9^

Studies have indicated several triggers for CVS episodes that are primarily psychological factors such as anxiety and stress.^8^, ^16^ This is consistent with Case 2, who reported that stress, inadequate sleep, and a change in life habits were triggers of his CVS episodes. Case 1 reported no recognized trigger factors. We observed in our cases that complete symptom resolution occurred between episodes, which is a major diagnostic criteria of CVS.^1^

The significant number of misdiagnoses in our paper highlights the importance of increasing the recognition and consideration of this disorder by healthcare personnel in patients with recurrent episodes of nausea and vomiting with symptom‐free intervals.^16^ While CVS has non‐specific symptoms and diagnostic findings, a number of cases have presented with non‐stereotypic manifestations, which can make the diagnosis and management more challenging.^17^ Case 2 was repeatedly misdiagnosed, which led to a three‐year delay in making the correct diagnosis. This is worthy of serious consideration.

The management of such cases should begin with a comprehensive patient history and physical and neurological examinations, as well as lab and further diagnostic tests and imaging to assess any alarming symptoms and to rule out possible gastrointestinal, neurological, immunological, and metabolic disorders.^10^ If none of the results are able to explain the vomiting and underlying organic causes can be ruled out, a cyclic pattern of vomiting that fulfills the diagnostic criteria should lead to a final diagnosis of CVS.^2^

The treatment of CVS is generally divided into following categories: (a) life‐style modification; (b) abortive therapy (during prodromal phase to prevent episodes); (c) supportive therapy during episodes and; (d) prophylactic therapy (daily, preventive treatment to prevent further prodromal and vomiting episodes).^2^ The primary therapeutic goals are to reduce the frequency and severity of the episodes and improve the patient's quality of life and functionality.^2^

A key element in management of CVS is a biopsychosocial approach and development of a good relationship with the patient and the patient's family.^16^ It is very important to provide sufficient information about the disorder to increase their cooperation and follow‐up and improve the therapeutic outcome.^16^

In the first step of treatment, life‐style modification includes avoidance of triggers, stress management, good sleep hygiene, and a healthy diet.^2^, ^18^ Moreover, treatment of the vomiting episodes is necessary. Abortive medications include anti‐emetics (5ht_3_‐receptor antagonists, antihistamines, and phenothiazine), anxiolytics (benzodiazepines), antimigraine (5‐HT1D agonist), and NSAIDs.^10^ Opioids might have efficacy but should be reserved for intractable cases. The most important point in both of our cases was that only rectal or intravenous diazepam was effective in improving the patient symptoms during the emetic phase, and that other analgesic and antiemetic medications had very limited efficacy.

Cheung et al.^19^ reported a case of an adult with CVS who was unresponsive to first‐line and second‐line medications, but whose symptoms dramatically resolved after treatment with ketamine. Most guidelines for management of the emetic phase of CVS have suggested the use of a benzodiazepine, mainly IV lorazepam.^13^, ^20^

To the best of our knowledge, this is the first study to report the effectiveness of rectal and intravenous diazepam for the emetic phase of CVS. It is suggested for future researchers and clinicians to study and report on the effectiveness of this agent over other medications for the emetic phase. This is important because, in an urgent situation where there is no access to IV administration or consideration of less invasive medications, the use of rectal diazepam could be a good therapeutic choice that is very effective and can be highly recommended.

Supportive therapy during the emetic phase consists of a combination of hydration, anti‐emetics (5‐HT3 receptor antagonists, phenothiazines, D2 receptor antagonists, antihistamines), anxiolytics (benzodiazepines), analgesics (NSAIDs, opioids), antimigraine (5‐HT1B/1D agonist), and gastric‐acid suppressants (proton pump inhibitors, H_2_ receptors antagonists).^10^ Moreover, the prophylactic medication can require a combination of tricyclic antidepressants, beta blockers, antihistamines, serotonin inhibitors, anti‐anxiety medication, and management of comorbid symptoms.^10^

The interesting and important point for reporting these two cases was the significant response to the use of 75 mg of cinnarizine every night for prophylaxis. As stated, antihistamines, mainly diphenhydramine, are among the suggested prophylactic medications for CVS.^21^ Cinnarizine has been reported for use as a prophylactic medication for migraine headache^22^, ^23^ and migraine associated vertigo.^24^ In both of our cases, medications such as nortriptyline, amitriptyline, propranolol, and sodium valproate were ineffective and did not considerably decrease the severity, frequency, or duration of the vomiting episodes, but cinnarizine could significantly improve the symptoms. To the best of our knowledge this is the first study to report on the efficacy and usage of cinnarizine for prophylaxis of CVS episodes. It is suggested that clinicians consider prescribing cinnarizine as a maintenance medication for CVS, especially for refractory cases. Psychological interventional protocols including ventilation techniques and healthy coping mechanisms have also been found to be effective for improvement of CVS symptoms in almost all phases.^25^

Regarding the prognosis of CVS, studies have shown complete symptom resolution among 61% of CVS children within 1 year of the onset of treatment, while 39% continued to experience CVS attacks and 40% of those who reported resolution of CVS symptoms continued to complain of somatic symptoms including headache and abdominal pain.^11^, ^16^ Thus, about one‐third of patients continue to experience CVS episodes despite receiving optimal medications.^11^, ^16^ There is a significant burden and disability associated with CVS because of hospitalization and work‐related and school absenteeism.^16^ Although CVS symptoms are debilitating, the vomiting resolves in 60% of cases quickly after diagnosis and treatment.^16^

## 4 CONCLUSION

We have reported on two cases of refractory CVS attacks in patients who were not responsive to recommended medications for acute phase and prophylaxis. We highlight the effectiveness of the use of rectal diazepam during the emetic phase over other established medications to abort symptoms. This approach is less invasive than IV administration and is highly recommended under urgent conditions where there is no access to IV administration or when less painful therapeutic methods are required. More importantly, this paper emphasizes the effectiveness of cinnarizine as a prophylactic agent. This has not been reported previously for the maintenance of CVS. Future case–control studies are suggested to better evaluate the efficacy of these two medications for the treatment of CVS.

## AUTHOR CONTRIBUTIONS

**Mansoureh Togha:** Conceptualization; data curation; formal analysis; funding acquisition; investigation; methodology; project administration; resources; software; supervision; validation; writing – original draft; writing – review and editing. **Mahsa Babaei:** Data curation; formal analysis; investigation; methodology; project administration; software; writing – original draft; writing – review and editing. **Melika Jameie:** Investigation; methodology; project administration; writing – original draft; writing – review and editing.

## FUNDING INFORMATION

None of the authors received any funding for this submission.

## CONFLICT OF INTEREST STATEMENT

The authors have no conflict of interests in this submission.

## ETHICS STATEMENT

All content of this research adheres to the ethical guidelines developed by the Committee on Publication Ethics (COPE) during the 2nd World Conference on Research Integrity in Singapore in 2010. All parts of this study meets the Code of Conduct (the Ethics Code) and adheres to the legal requirements of the study country, Iran.

## CONSENT

Written and verbal informed consent have been properly obtained from the patients and their guardians.

## Data Availability

Data supporting this paper are available via contacting the corresponding author.
